# Supplementary material for: Conformal Swallowing Accelerometry: Reimagining the Acquisition and Characterization of Swallowing Mechano-Acoustic Signals
Source: Sensors (Basel). 2025 Dec 4;25(23):7396. doi: 10.3390/s25237396 (PMC12694341; doi:10.3390/s25237396)
Supplement: Supplementary file 1 [file sensors-25-07396-s001.zip › sensors-3967962-supplementary/Table S1.pdf]

Table S1. Inter-trial agreement across volumes.

variable	channel	volume	ICC	*F*	df1	df2	*p*
peak intensity	1	5	0.524	4.305	12	24	< 0.01
		10	0.751	10.065	12	24	< 0.001
	2	5	0.377	2.818	12	24	< 0.054
		10	−0.009	0.973	12	24	0.499
	3	5	0.311	2.354	12	24	< 0.05
		10	0.650	6.561	12	24	< 0.001
	5	5	0.145	1.507	12	24	0.189
		10	0.625	6.007	12	24	< 0.001
	6	5	0.233	1.910	12	24	0.086
		10	0.427	3.234	12	24	< 0.01
	7	5	0.658	6.766	12	24	< 0.001
		10	0.334	2.502	12	24	< 0.05
Peak frequency	1	5	−0.095	0.740	12	24	0.701
		10	0.557	4.778	12	24	< 0.001
	2	5	0.139	1.484	12	24	0.198
		10	−0.098	0.733	12	24	0.707
	3	5	0.265	2.084	12	24	0.061
		10	0.449	3.447	12	24	< 0.01
	5	5	0.160	1.570	12	24	0.168
		10	0.068	1.219	12	24	0.326
	6	5	0.012	1.037	12	24	0.449
		10	−0.024	0.929	12	24	0.536
	7	5	0.087	1.287	12	24	0.288
		10	0.117	1.398	12	24	0.233
