# Supplementary material for: Conformal Swallowing Accelerometry: Reimagining the Acquisition and Characterization of Swallowing Mechano-Acoustic Signals
Source: Sensors (Basel). 2025 Dec 4;25(23):7396. doi: 10.3390/s25237396 (PMC12694341; doi:10.3390/s25237396)
Supplement: Supplementary file 1 [file sensors-25-07396-s001.zip › sensors-3967962-supplementary/Table S2.pdf]

Table S2. Inter-channel agreement across volumes.

variable	comparison	volume	ICC	*F*	df1	df2	*p*
peak intensity	1 vs 5	5	0.500	2.971	38	38	< 0.001
		10	0.536	3.410	38	38	< 0.001
	2 vs 6	5	0.404	2.388	38	38	< 0.01
		10	−0.026	.951	38	38	0.562
	3 vs 7	5	0.605	4.544	38	38	< 0.001
		10	0.639	4.593	38	38	< 0.001
	left	5	0.352	2.646	38	76	< 0.001
		10	0.023	1.072	38	76	0.391
	right	5	0.075	1.248	38	76	0.205
		10	0.157	1.571	38	76	< 0.05
	gross	5	0.185	2.379	38	190	< 0.001
		10	0.055	1.349	38	190	0.100
peak frequency	1 vs 5	5	0.262	1.707	38	38	0.052
		10	0.141	1.356	38	38	0.176
	2 vs 6	5	−0.024	.954	38	38	0.557
		10	−0.079	.855	38	38	0.684
	3 vs 7	5	0.164	1.418	38	38	0.143
		10	0.045	1.094	38	38	0.392
	left	5	0.176	1.647	38	76	< 0.05
		10	0.005	1.014	38	76	0.468
	right	5	0.104	1.351	38	76	0.133
		10	0.154	1.554	38	76	0.052
	gross	5	0.134	1.934	38	190	< 0.01
		10	0.061	1.395	38	190	0.077
