# Supplementary material for: Conformal Swallowing Accelerometry: Reimagining the Acquisition and Characterization of Swallowing Mechano-Acoustic Signals
Source: Sensors (Basel). 2025 Dec 4;25(23):7396. doi: 10.3390/s25237396 (PMC12694341; doi:10.3390/s25237396)
Supplement: Supplementary file 1 [file sensors-25-07396-s001.zip › sensors-3967962-supplementary/Table S3.pdf]

Table S3. Tabulated comparison of agreement and correlation of peak values and their respective time across volumes.

			agreement					correlation	
comparison	variable	volume	ICC	*F*	df1	df2	*p*	τ	*p*
1 vs 5	peak intensity	5	0.500	2.971	38	38	< 0.001	0.547	< 0.001
		10	0.536	3.41	38	38	< 0.001	0.619	< 0.001
	peak intensity time	5	0.977	85.391	38	38	< 0.001	0.876	< 0.001
		10	0.967	6.86	38	38	< 0.001	0.843	< 0.001
	peak frequency	5	0.262	1.707	38	38	0.052	0.382	< 0.001
		10	0.141	1.356	38	38	0.176	0.282	< 0.05
	peak frequency time	5	0.976	82.71	38	38	< 0.001	0.895	< 0.001
		10	0.982	114.307	38	38	< 0.001	0.884	< 0.001
2 vs 6	peak intensity	5	0.404	2.388	38	38	< 0.01	0.271	< 0.05
		10	−0.026	0.951	38	38	0.562	0.287	< 0.01
	peak intensity time	5	0.960	48.868	38	38	< 0.001	0.827	< 0.001
		10	0.946	38.174	38	38	< 0.001	0.841	< 0.001
	peak frequency	5	−0.024	0.954	38	38	0.557	0.080	0.475
		10	−0.079	0.855	38	38	0.684	0.185	0.097
	peak frequency time	5	0.988	162.537	38	38	< 0.001	0.908	< 0.001
		10	0.944	33.759	38	38	< 0.001	0.838	< 0.001
3 vs 7	peak intensity	5	0.605	4.544	38	38	< 0.001	0.287	< 0.01
		10	0.639	4.593	38	38	< 0.001	0.552	< 0.001
	peak intensity time	5	0.931	27.33	38	38	< 0.001	0.787	< 0.001
		10	0.955	47.199	38	38	< 0.001	0.822	< 0.001
	peak frequency	5	0.164	1.418	38	38	0.143	0.242	< 0.05
		10	0.045	1.094	38	38	0.392	0.347	< 0.01
	peak frequency time	5	0.994	351.771	38	38	< 0.001	0.919	< 0.001
		10	0.993	275.191	38	38	< 0.001	0.919	< 0.001
